# A systematic review of evidence-based practice implementation in drug and alcohol settings: applying the consolidated framework for implementation research framework

**DOI:** 10.1186/s13012-021-01090-7

**Published:** 2021-03-04

**Authors:** Eva Louie, Emma L. Barrett, Andrew Baillie, Paul Haber, Kirsten C. Morley

**Affiliations:** 1 Sydney School of Medicine, Faculty of Medicine and Health, The University of Sydney, Sydney, NSW 2006 Australia; 2 Edith Collins Centre (Alcohol, Drugs and Toxicology), Sydney Local Health District, Sydney, NSW Australia; 3 The Matilda Centre for Research in Mental Health and Substance Use, Faculty of Medicine and Health, The University of Sydney, Sydney, NSW Australia; 4 Faculty of Health Sciences, The University of Sydney, Sydney, NSW Australia; 5 Drug Health Services, Royal Prince Alfred Hospital, Sydney, NSW Australia

**Keywords:** Implementation, Alcohol, Substance use, Addiction, Systematic review

## Abstract

**Background:**

There is a paucity of translational research programmes to improve implementation of evidence-based care in drug and alcohol settings. This systematic review aimed to provide a synthesis and evaluation of the effectiveness of implementation programmes of treatment for patients with drug and alcohol problems using the Consolidated Framework for Implementation Research (CFIR).

**Methods:**

A comprehensive systematic review was conducted using five online databases (from inception onwards). Eligible studies included clinical trials and observational studies evaluating strategies used to implement evidence-based psychosocial treatments for alcohol and substance use disorders. Extracted data were qualitatively synthesised for common themes according to the CFIR. Primary outcomes included the implementation, service system or clinical practice. Risk of bias of individual studies was appraised using appropriate tools. A protocol was registered with (PROSPERO) (CRD42019123812) and published previously (Louie et al. Systematic 9:2020).

**Results:**

Of the 2965 references identified, twenty studies were included in this review. Implementation research has employed a wide range of strategies to train clinicians in a few key evidence-based approaches to treatment. Implementation strategies were informed by a range of theories, with only two studies using an implementation framework (Baer et al. J Substance Abuse Treatment 37:191-202, 2009) used Context-Tailored Training and Helseth et al. J Substance Abuse Treatment 95:26-34, 2018) used the CFIR). Thirty of the 36 subdomains of the CFIR were evaluated by included studies, but the majority were concerned with the *Characteristics of Individuals* domain (75%), with less than half measuring *Intervention Characteristics* (45%) and *Inner Setting* constructs (25%), and only one study measuring the *Outer Setting* and *Process* domains. The most common primary outcome was the effectiveness of implementation strategies on treatment fidelity. Although several studies found clinician characteristics influenced the implementation outcome (40%) and many obtained clinical outcomes (40%), only five studies measured service system outcomes and only four studies evaluated the implementation.

**Conclusions:**

While research has begun to accumulate in domains such as Characteristics of Individuals and Intervention Characteristics (e.g. education, beliefs and attitudes and organisational openness to new techniques), this review has identified significant gaps in the remaining CFIR domains including organisational factors, external forces and factors related to the process of the implementation itself. Findings of the review highlight important areas for future research and the utility of applying comprehensive implementation frameworks.

**Supplementary Information:**

The online version contains supplementary material available at 10.1186/s13012-021-01090-7.

Contributions to the literature

- This systematic review is the first to apply the comprehensive Consolidated Framework for Implementation Research (CFIR) to synthesise and evaluate the effectiveness of implementation programmes in the treatment of patients with drug and alcohol problems.
- Most studies in this field focus on ***Characteristics of Individuals*** **or** ***Intervention Characteristics***, with less consideration of the remaining CFIR domains including organisational factors, external forces and factors related to the implementation process.
- The most common primary outcome was the effectiveness of implementation strategies on treatment fidelity and only 25% of studies measured service system outcomes.

## Introduction

There is a lack of evidence-based treatment approaches being practised in drug and alcohol settings [1–3]. Evidence-based treatments including addiction medications, psychosocial therapies or integrated services are estimated to have been provided by no more than 25% of community services treating substance use disorders (SUDs) or co-occurring mental health disorders [4]. Furthermore, known effective treatments for SUDs are not routinely practised [3, 5, 6]. Bridging this gap requires a systematic assessment of the barriers that exist at multiple levels of healthcare delivery including the patient level, the provider level and the organisational level, and an associated plan for overcoming these barriers [7]. Bridging factors can be identified that work between system and organisational levels or interorganisational networks [8]. This would provide valuable information for clinicians and treatment services designed to ultimately address the pervasive harms associated with drug and alcohol use disorders.

Identifying evidence-based interventions for SUDs rather than developing an evidence-based implementation strategy appears to have previously received more focus [9] whereby research is generally conducted under controlled conditions that may not translate when implemented in practice settings. To this degree, the knowledge accumulated by the field of implementation science has informed the process of effectively implementing innovations and understanding treatment outcomes as distinct from implementation outcomes [10, 11]. Despite the high burden of disease [12] and the sizable gap between research and practice, the addictions field is grossly underrepresented within implementation science [4]. The application of implementation science to the implementation of evidence-based treatment of SUDs is therefore a priority.

Several frameworks have been developed appropriate for public sector services that have high utility in formulating implementation strategies, identifying appropriate assessments and assessing determinants and mechanisms (e.g. [13, 14], CFIR, 15 below). In the specific context of SUD research, the Consolidated Framework for Implementation Research [15] has been suggested to be an appropriate taxonomy [11]. The CFIR includes five domains of influence derived from a consolidation of the plethora of terms and concepts generated by implementation researchers: (1) intervention characteristics (e.g. evidence strength and quality, adaptability), (2) outer setting (e.g. patient needs and resources, external policies and incentives), (3) inner setting (e.g. implementation climate, readiness for implementation), (4) individuals involved (e.g. self-efficacy, knowledge and beliefs about the intervention), and (5) the implementation process (e.g. engaging members of the organisation, executing the innovation). A particular strength of the CFIR is the way in which it assists with differentiating the core components from the adaptive components of the intervention [3, 16], provides a platform for formative evaluation in implementation research and allows for the development and evaluation of models designed to predict the determinants of implementation outcomes and sustainability in a given context [11]. Another potential use for the CFIR is the assessment of how comprehensive an implementation strategy has been [17, 18]. Due to the relationship between the domains of the CFIR and the implementation outcomes, it has been categorised as a “determinant framework” [19]. As one of many determinant frameworks in the implementation research literature, the CFIR is distinguished by its comprehensive approach to synthesising implementation research. The incorporation of inner and outer setting domains in addition to clinician characteristics is of particular importance in the drug and alcohol field, which operates within these contexts. These attributes, as well as its utility in previous reviews and the SUD context, have made it the most appropriate evaluation framework for this review.

There are considerably less empirical evaluations of implementation strategies in SUD settings [20] than those found in the broader field of health care [17]. Reviews conducted to date have primarily been concerned with prevention (e.g. [21, 22]), treatment efficacy (e.g. [23, 24]) and specific interventions (e.g. [25, 26]). Where implementation strategies have been identified, the focus of the review has been on strategies addressing specific factors (e.g. [27]) or relationships between factors (e.g. [28]) related to implementation outcomes, but there has not been a comprehensive account of implementation effectiveness. One previous review of the implementation of SUD treatment [25] specifically focused on one type of intervention (integrated care). A thorough synthesis of implementation strategies in the SUD field in general, using an appropriate framework such as the CFIR is required to guide the design of translational research programmes to improve implementation of evidence-based care in drug and alcohol settings.

The objectives of this systematic review are thus to synthesise and evaluate the effectiveness of implementation programmes for psychosocial treatment of patients with drug and alcohol problems with regard to the five domains of influence outlined by the CFIR framework.

## Methods

The present review is being reported in accordance with the reporting guidelines of the Preferred Reporting Items for Systematic Reviews and Meta-Analyses Protocols (PRISMA-P) statement [29], see Additional file 1. A protocol was registered within the International Prospective Register of Systematic Reviews (PROSPERO) (registration number: CRD42019123812) and published previously [30].

### Eligibility criteria

Criteria for considering studies for this review were classified by:

#### Population

In order to meet inclusion criteria, studies had to involve an evaluation of implementation strategies used to transfer an evidence-based psychosocial treatment or treatment guideline into clinical practice in drug and alcohol settings. Implementation strategies were defined as an integrated set of methods or techniques that facilitate the adoption, implementation and sustainability of best practice [31]. Examples of discrete categories of implementation strategies included in this review have been most clearly articulated by Powell et al. [32]. Psychosocial treatments included any attempt to affect change in patients’ substance use through behaviour, cognition, affect, interpersonal relationships or environment (e.g. employment, housing). Participants in these studies included any clinician providing psychosocial interventions to patients accessing outpatient or inpatient drug and alcohol services. “Clinician” was defined as an individual employed to implement change in patients’ substance use using psychosocial treatments exclusively. As such, studies were excluded from the review if they focused on the development of psychometric instruments, drugs in sport, harm prevention or community awareness.

#### Intervention

To be eligible, the psychosocial intervention had to be evidence-based and provide clear recommendations for practice. Studies were excluded if they involved physiological, pharmacological (except where concurrent medication was provided but was not part of the study intervention primarily being examined or implemented), or education-based interventions. Information including the nature of desired change, strategies employed, source of the intervention, mode of delivery (individual or group), identification of who delivered the intervention, and the timing, duration and frequency of the intervention had to be stated clearly. Only ethically approved studies were considered.

#### Comparator and study design

Only studies with a comparison group were included. Comparisons could be made before and after the administration of the intervention, between two or more forms of intervention, or between different types of intervention(s) (or no intervention). We included randomised controlled trials (RCTs), non-randomised controlled trials, observational studies including before-and-after studies, and time series analyses.

#### Outcomes

Primary study outcomes were adapted from previous studies [9, 33], and included implementation, service system or clinical practice. Specifically, outcomes covered categories such as fidelity, attitudes towards or satisfaction with the intervention, adoption, appropriateness of the intervention to the target population, implementation costs, the feasibility of the intervention within the setting and the sustainability of the intervention after implementation [33]. The length of post-intervention follow-up period had to be specified and any possible ceiling effects identified. Outcomes needed to be related to the effectiveness of the implementation process, as distinct from the efficacy of the intervention itself.

#### Setting

Since drug and alcohol inpatient and outpatient treatment settings that provide counselling services to patients are the focus of the review, settings such as primary care, criminal justice or those investigating cross-cultural factors were excluded from the review.

### Information sources

The following electronic databases were searched (from inception to April 2020): PubMed/MEDLINE, Cochrane Library, PsycINFO, Web of Science, and CINAHL. Reference searches of relevant reviews and articles were also conducted. Similarly, a grey literature search was done with help of Google and the Grey Matters tool which is a checklist of health-related sites organised by topic. The tool is produced by the Canadian Agency for Drugs and Technologies in Health (CADTH) [34].

### Search strategy

The search included all relevant peer-reviewed studies. The search was conducted across 4 relevant concepts (see draft strategy in Additional file 2): (1) implementation, (2) evidence-based practices, (3) drug and alcohol service setting and (4) eligible research designs. The MEDLINE search strategy is available in Additional file 2.

### Selection and data extraction

Two reviewers independently screened all articles identified from the search. First, titles and abstracts of articles returned from initial searches were screened based on the eligibility criteria outlined above. Second, full texts were examined in detail and screened for eligibility. Third, references of all considered articles were hand-searched to identify any relevant report missed in the search strategy by two reviewers independently. Any disagreement between reviewers was resolved by discussion to meet a consensus. EndNote version X9 (Clarivate Analytics) was used to manage all records.

Two researchers extracted data and organised it into variables based on the Cochrane Effective Practice and Organisation of Care (EPOC) Data Abstraction Form (e.g. clinical interventions, strategies, outcomes, and results), the conceptual model of Proctor et al. [9] (implementation, service system and clinical outcomes), information about any specific implementation frameworks used and a checklist of items aligned with the domains and subdomains of the CFIR (i.e. subdomains associated with *intervention characteristics, outer setting, inner setting, characteristics of individuals*, and the implementation *process*; see Table 1). This method was used effectively in two previous reviews [18, 35] as a means of categorising the types of implementation strategies addressed by each of the studies included in the review.

**Table 1:** Brief description of CFIR constructs

Domain	Construct	Description
INTERVENTION CHARACTERISTICS	Intervention Source	Understanding about whether the intervention was developed internally or externally
	Evidence Strength and Quality	Beliefs about the quality and validity of evidence for the intervention and whether it will achieve the intended outcomes
	Relative Advantage	The advantages of implementing the intervention compared to other possible alternatives
	Adaptability	How readily the intervention can be adapted to the specificities of the local context
	Trialability	Whether the intervention can be piloted on a small scale initially and undone if necessary
	Complexity	How difficult the intervention is to implement (duration, scope, departure from norm, number of steps required)
	Design Quality and Packaging	How well the intervention was bundled, presented and assembled
	Cost	The cost of using and implementing the intervention (investment, supply and opportunity costs)
OUTER SETTING	Patient Needs and Resources	How well the organisation prioritises understanding barriers and facilitators to meeting patient needs
	Cosmopolitanism	How well networks have been established with external organisations
	Peer Pressure	Whether pressure is felt to implement the intervention in order to compete with fellow organisations, who have already done so
	External Policy and Incentives	Externally imposed (policy, regulations, government) strategies (e.g. guidelines, benchmark reporting) designed to increase use of the intervention
INNER SETTING	Structural Characteristics	The age, maturity and size and social structure of the organisation
	Networks and Communications	The effectiveness of social networks and communication (formal and informal)
	Culture	Organisational norms, values and assumptions
	Implementation Climate	The organisation’s capacity for making the necessary changes, whether individuals within the organisation are receptive to change, and how well the organisation supports, rewards and anticipates use of the intervention
	- Tension for Change	Whether there is a perception that change is necessary
	- Compatibility	How well the underlying meaning and values of the intervention complement existing norms, values, opinions about risk, and workflows and systems
	- Relative Priority	The degree of importance given to the implementation compared to other competing priorities
	- Organisational Incentives and Rewards	These may include reaching shared goals, performance reviews, promotions, pay increases, recognition
	- Goals and Feedback	How well goals are established and whether meaningful feedback is provided along the way
	- Learning Climate	A positive learning climate involves: leaders who accept fault and encourage team input; team members who feel essential, valued and knowledgeable; a psychologically safe context for uptake of the intervention; and time and space to reflect on and evaluate progress
	Readiness for Implementation	Whether the organisation demonstrates a tangible and immediate commitment to implement the intervention
	- Leadership Engagement	How committed, involved and accountable leaders and managers are to implementation
	- Available Resources	Whether adequate resources have been allocated to the implementation and sustainment of the intervention (e.g. money, training, education, space, time)
	- Access to Knowledge and Information	The availability of information and knowledge about the intervention that is easy to understand and incorporate into work tasks
CHARACTERISTICS OF INDIVIDUALS	Knowledge and Beliefs about the Intervention	Attitudes related to the value of the intervention, and knowledge of the evidence and principles behind the intervention
	Self-efficacy	Whether the individual believes they are capable of performing tasks required to achieve implementation goals
	Individual Stage of Change	Phase of change from pre-contemplation to skilled, enthusiastic and sustained implementation of the intervention
	Individual Identification with Organisation	The individual’s perception of the organisation, their place within it, and their commitment to it
	Other Personal Attributes	Other personal factors influencing the implementation (intellectual ability, motivation, values, competence, learning style
PROCESS	Planning	How well the preliminary methods of behaviour and implementation tasks are developed and how appropriate they are
	Engaging	Execution of strategies (social marketing, education, training) for attracting and involving the right people
	Opinion Leaders	Individuals who have influence over their colleagues’ attitudes and beliefs about the intervention
	Formally Appointed Internal Implementation Leaders	Individuals who have been given responsibility for implementing the intervention within the organisation
	Champions	Individuals who elect to support, market and assist with overcoming resistance to the implementation
	External Change Agents	Individuals from an external entity who have a formal role in promoting the implementation of the intervention
	Executing	Whether the implementation is carried out as planned
	Reflecting and Evaluating	Regular individual and team debriefing about the progress and experience of the implementation, and the nature and quality of quantitative and qualitative feedback used

### Risk of bias of individual studies

All included studies were critically evaluated by two researchers independently using the Revised Cochrane risk-of-bias tool (RoB 2) [22]. The RoB 2 provides a systematic assessment across five domains of bias (the randomisation process, deviations from intended interventions, missing outcome data, measurement of the outcome, and selection of the reported results) to assess quality of the article per outcome. For cluster-randomised studies, an additional domain was used when assessing the randomisation process. Trial registries were also checked to determine the integrity of reported outcome measures and statistical methods. The grey literature search also assisted with identifying publication bias.

### Data synthesis

Included studies did not have sufficient characteristics for a meta-analysis and therefore a narrative synthesis was performed. The main methods of synthesis involved tabulation using “meta-matrices” [36], textual descriptions, qualitative synthesis of themes [37] and content analysis to determine the frequency of categorised data [38]. The findings from the included articles were synthesised using the CFIR framework.

## Results

### Search results

**Fig. 1:**
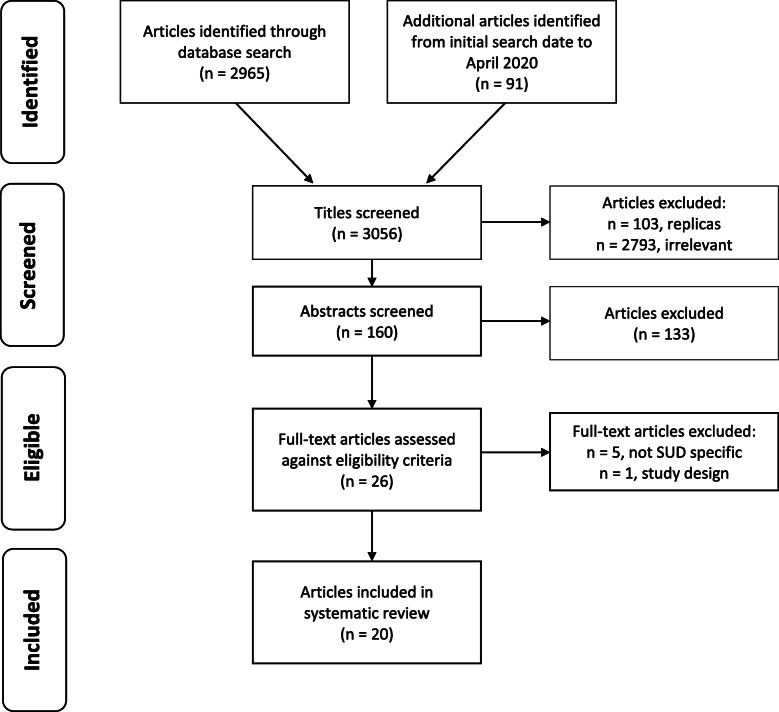
Study Selection

As displayed in the flowchart (Fig. 1), the database search identified 2965 studies. After titles were screened, 159 studies were found to be relevant (103 of which were replicas). Abstracts of the remaining studies were screened and 26 were found to meet inclusion criteria. Finally, full-text articles of these studies were assessed for eligibility and 19 were included in the review. An additional, identical search was conducted to capture any further relevant studies conducted between the time the first search was conducted until April 2020. This search identified 91 studies, one of which met eligibility criteria and was included in the review. An outline of the main features of included studies is provided in Table 2, including the type of innovation, guiding theories, strategies employed, study design, treatment setting, participant characteristics, study outcomes, CFIR domains evaluated, and the effectiveness of the implementation.

**Table 2:** Summary of included studies

	Type of Innovation	Implementation Theories, Models and Frameworks	Types of Strategies Evaluated	Design	Sample	Factors Evaluated	Effectiveness of Implementation
Baer et al. 2009 [39]	Motivational Interviewing (MI)	"Context Tailored Training” (CTT) Characteristics of Clinicians: tailoring the intervention to the specific context. An adaptation of Rollnick et al.’s [40] “context-bound” training.	CONTEXT Tailoring the intervention to the specific work context vs. 2-day workshop	Randomised trial	Participants: Gender: female (68%), Ethnicity: Caucasian(81%), Age: 42 years, Education: Bachelor’s degrees or more (68%), Experience: 4.8 years Treatment Setting: United States of America (USA), community-based, National Institute on Drug Abuse (NIDA)	Primary Outcomes: Fidelity to intervention Adherence to training Predictors of implementation: Clinician characteristics: demographics, perspectives on current work, beliefs about the origin and treatment of addictive behaviours Clinician Evaluation: satisfaction with training Acceptability and appropriateness: *Organisational Readiness for Change (ORCA* [41];*)* and Perception of Agency Support	Primary Outcome: CTT did not improve training outcomes, but mitigating factors found. Predictors of implementation: Clinician Characteristics: Higher education and lower endorsement of disease model beliefs Clinician Evaluation: Modest differences between conditions in satisfaction. Acceptability: Encouraging staff to do new things, higher self-efficacy and greater openness to new techniques
Carpenter et al. (2012) [42]	MI	Nil	TECHNOLOGY SUPERVISION Workshop plus tele-conferencing supervision vs. workshop plus standard tape-based supervision vs. workshop alone	Randomised trial	Participants: Education: Bachelor’s degree or more (69%), Therapeutic Orientation: Cognitive Behavioural Therapy (CBT) (79%), harm reduction (45%), Alcoholics Anonymous/Narcotics Anonymous (AA/NA) principles (32%), MI (10%), Treatment Setting: USA, community-based, NIDA	Primary Outcome: Fidelity to intervention Predictors of implementation: Clinician Characteristics: age, gender, ethnicity, counselling style, verbal and abstract reasoning skills	Primary Outcome: Clinician characteristics moderated the effect. Predictors of implementation: Clinician Characteristics: Less education, strong vocabulary and low average verbal abstract reasoning
Carroll et al. (2006)	MI	Nil	MULTIPLE Workshop and supervision (randomised to either MI training group or standard intake/ evaluation group)	Randomised trial	Participants: Gender: female (68%), Ethnicity: Caucasian (81%), Age: 42 years, Education: Bachelor’s degree or more (68%), Experience: 7 years Treatment Setting: USA, community-based, NIDA	Primary Outcome: Fidelity to the intervention Predictors of implementation: Clinician Characteristics: demographics, experience, counselling orientation, and clinical techniques Clinical Outcomes: Retention Substance use timeline follow back (TLFB) Predictors of clinical outcomes: Characteristics of Patients: demographics, legal system involvement	Primary Outcomes: Community-based clinicians achieve fidelity when provided training and supervision. Predictors of implementation: No significant findings Clinical Outcomes: MI training group had significantly better retention through the 28-day follow-up than those assigned to the standard intervention.
Decker and Martino (2013) [43]	MI	Rogers et al. [44]: individuals are more likely to adopt an intervention after they have an increased knowledge about it and then develop a more favourable attitude towards it.	MULTIPLE/ LOCAL EXPERT Self-study vs. workshop and supervision, vs. workshop and supervision from program-based trainers	Randomised trial	Participants: No information of whole sample at baseline Treatment Setting: USA, community-based, NIDA	Primary Outcome: Fidelity to the intervention Clinician Predictors of implementation: Clinician Characteristics: demographics, experience, treatment allegiance, recovery status, interest, confidence and commitment in using intervention.	Primary Outcome: No significant differences found. Predictors of implementation: Confidence was associated with increased competence in the use of advanced MI strategies.
Garner et al. (2012) [45]	The Adolescent Community Reinforcement Approach (A-CRA)	Nil	FINANCIAL INCENTIVE “Pay for Performance” (P4P) vs. controls	Cluster randomised trial	Participants: Gender: female (74%), Ethnicity: Caucasian (55%), Age: 36.5 years, Education: Master's Degree or higher (55%), Experience: 6.5 years Treatment Setting USA, community-based, funded by Substance Abuse and Mental Health Services (SAMHSA)	Primary Outcome: Fidelity to intervention Clinical Outcomes: Remission status Substance use	Primary Outcome: P4P therapists were significantly more likely to demonstrate A-CRA competence. Clinical Outcomes: Patients in the P4P condition were significantly more likely to receive target A-CRA. No significant differences between conditions with regard to patients' end-of-treatment remission status.
Gaume et al. (2014) [46]	Brief motivational intervention (BMI)	Nil	WORKSHOP ONLY vs. controls	Randomised Controlled Trial (RCT)	Participants: Gender: 'equally distributed', Experience: 8.3 years Treatment Setting: Switzerland, outpatient service, University Hospital	Predictors of implementation: Fidelity to intervention Clinician Characteristics: demographics, experience, experience in intervention, views of the intervention Self-report of effectiveness in implementing BMI Clinical Outcomes: Substance Use: a drinking composite score, usual drinks per drinking day, and frequency of binge drinking Predictors of Clinical Outcomes: Patient Characteristics: demographics	Predictors of implementation: Clinician Characteristics: Age and experience - young men with more experienced counsellors had significantly better outcomes than young men having had no intervention. Beliefs - Counsellors viewing themselves as more effective in delivering BMI and having higher belief in BMI efficacy also had clients with better outcomes. Clinical Outcomes: Significant decrease in alcohol use among the BMI group on all three drinking variables.
Helseth et al. (2018) [47]	Contingency Management (CM)	Consolidated Framework for Implementation Research [11] Rogers’ [48]: Diffusion of Innovations theory	MULTIPLE/ LOCAL EXPERT Treatment as usual (TAU) vs. TAU plus access to a technology transfer specialist plus innovation champion plus role-specific training in the change process ["Science to Service Laboratory" (SSL)]	Controlled before-and-after study	Participants: Gender: female (68%), Ethnicity: ‘minority’ (23%), Caucasian (77%), Experience: 60% had 3+ years, Education: Bachelor’s degree or more (23%), Treatment Setting: USA, community-based settings	Primary Outcome: Adoption of intervention Predictors of implementation: Clinician Characteristics: demographics, experience, caseload Clinician Evaluation: *Provider Attribute Scale* (*PAS* [49];*)* Acceptability and appropriateness: *ORCA* [41]	Primary Outcome: SSL significantly increased CM adoption. Predictors of implementation: Acceptability and appropriateness: Intervention Characteristic - *Compatibility* had a negative effect on CM adoption that was attenuated among SSL-providers.
Johnson et al. (2002) [50]	Therapeutic community (TC) drug treatment - drug abuse treatment (DAT) services	"Therapeutic community treatment theory" [51]: devised for the Drug Abuse Treatment Training Experiment. "Program Theory" [52]: Johnson et al. [53] demonstrated how a pro-gram theory can be tested in the substance abuse field.	BOOSTER TRAINING SESSIONS 6 weeks basic training vs. 8 weeks basic training plus booster sessions - theoretically grounded Managing Organisational Change (MOC) course.	A subject-by-trial split-plot design with repeated measures. Randomised trial	Participants: No information of whole sample at baseline Treatment Setting: Peru, Drug Abuse Treatment organisations, USA Department of State contract	Primary Outcome: Fidelity to intervention Predictors of implementation: Clinician Characteristics: demographics, experience, prior training and exposure to intervention, level of stress, cognitive and affective learning Clinician evaluation: training appraisals, trainer competency, curriculum content, classroom environment, and cultural sensitivity Appropriateness, Penetration: organisational characteristics including TC certification status, description of service Clinical Outcomes: Retention Service System Outcomes: Location, entry criteria, types of services offered, client to staff ratio, staff turnover, record data quality	Primary Outcomes: The basic training in combination with the MOC increased the magnitude of effects. Predictors of implementation: Clinician Characteristics: some aspects of ‘affective learning’ established and maintained. Clinician Evaluation: nearly all participants gave positive appraisals of the trainers, the training content and methods, the training environment, and the cultural sensitivity. Penetration: DAT training influenced organisational decisions to implement TC methods with fidelity in the booster training session group. Clinical and Service System Outcomes: no significant findings
Larson et al. (2013) [54]	Web based CBT course for addiction counsellors named TEACH-CBT (Technology to Enhance Addiction Counselor Helping)	Nil	TECHNOLOGY Online CBT course vs. training with treatment manual	Randomised trial	Participants: No information of whole sample at baseline Treatment Setting: USA, Outpatient and residential facilities, NIDA	Primary Outcome: Fidelity to intervention Predictors of implementation: Clinician Characteristics: demographics, prior training, exposure to the adoption of new techniques, attitudes towards evidence-based treatments (EBTs), intervention strategies, barriers, and knowledge Feasibility: unit size	Primary Outcome: Web-course participation did not increase fidelity relative to training with treatment manual Predictors of implementation: Feasibility: Unit size – web course training achieved higher fidelity in larger addiction units and training with a treatment manual achieved higher fidelity in the smaller agencies.
Liddle et al. (2010) [55]	Multi-dimensional family therapy (MDFT)	Simpson [56]: systemically-oriented dissemination models, and the evaluation of these efforts in multiple domains, including organisational, clinician and client outcomes.	CONTEXT Collaboration with staff, administration and patient outcomes (design implies that they were their own controls)	Interrupted time series design	Participants: Gender: female (80%), Ethnicity: Hispanic (50%), African American (20%), White (20%), Haitian (10%), Education: Bachelor’s and above (70%) Treatment Setting: Florida USA, Adolescent Day Treatment Program, University of Miami Medical School/Jackson Memorial Hospital	Primary Outcomes: Fidelity to intervention Adherence to intervention approach Predictors of implementation: Penetration: program level changes *Community-Oriented Programs Environment Scale* [57] Clinical Outcomes: Substance use (TLFB and urine screens) Emotional and Behavioural symptoms (*Child Behaviour Checklist* and *Youth Self Report* [58]*)*	Primary Outcome: Fidelity to the intervention was obtained following the intervention, and changes were sustained over time. Predictors of implementation: Penetration: Program environment more controlled, more practical and useful approach, clearer expectations, greater autonomy. Clinical Outcomes: Increased abstinence. Reduction in internalising and externalising behaviour.
Martino et al. (2008) [59]	Motivational Enhancement Therapy (MET)	Nil	MULTIPLE/ LOCAL EXPERT Workshop, supervision, local experts vs. counselling as usual	RCT	Participants: Gender: female (60%), Age: 39 years, Ethnicity: Caucasian (77%), Education: Masters’ degree (43%), Experience: 8.1 years, Treatment Setting: USA, Outpatient (non-methadone), NIDA	Primary Outcome: Fidelity to intervention Predictors of implementation: Clinician characteristics: experience, education, and commitment to empirically supported therapies Clinical Outcomes: Change in motivation Substance Use (self-reports TLFB and urine samples)	Primary Outcome: Community program clinicians can be trained to administer MET with fidelity. Predictors of implementation: No significant findings. Clinical Outcome: Greater fidelity was associated with increases in client motivation and some positive client treatment outcomes.
Martino et al. (2011) [60]	MI	Nil	CONTEXT Train-the-trainer vs. self-study	Randomised trial	Participants: Gender: female (65%), Ethnicity: Caucasian (83%), Education: Master’s degree (50%) Treatment Setting: USA, Outpatient programs	Primary Outcome: Fidelity to intervention	Primary Outcomes: The train-the-trainer group increased fidelity to the intervention at different assessment points comparted to the self-study group. Predictors of implementation: Gains required a substantial amount of training and implementation resources. Clinicians may need more supervision over time.
Martino et al. (2016) [61]	MI	Nil	SUPERVISION A more cost-effective supervision approach – Motivational Interviewing Assessment: Supervisory Tools for Enhancing Proficiency (MIA:STEP) vs. supervision as usual	RCT (hybrid type 2)	Participants: Gender: female (79%) Age: 41 years, Ethnicity: Caucasian (65%), Hispanic, (20%), African American, (14%), other (1%), Education: Bachelor’s Degrees or more (72%), Experience: 8 years Treatment Setting: USA, Outpatient Programs, non-for-profit	Primary Outcomes: Fidelity to intervention Supervision integrity *Supervision Adherence and Competence Scale* Implementation Outcome: Cost of the intervention Clinical Outcomes: Treatment Retention Substance Use (TLFB, breathalysers and urine screening) Treatment utilisation (of alternate services)	Primary Outcomes: MIA: STEP increased fidelity significantly more than supervision as usual. Supervision delivery and integrity - significantly better MIA: STEP. Implementation Outcome: Cost - MIA: STEP substantially more expensive compared to usual supervisory practices. Clinical Outcomes: similar rates of attendance, program retention, abstinence between groups.
Meier et al. (2015) [62]	Integrated Cognitive Behavioural Therapy (ICBT) or Individual Addiction Counselling (IAC).	Nil	MULTIPLE Manual, workshop, supervision vs. control	RCT	Participants: Gender: female (82%), Age: 44 years, Ethnicity: Caucasian (100%), Education: Bachelor’s Degree or more (100%), Experience: 7 years Treatment Setting: USA, community outpatient, not-for-profit	Primary Outcome: Fidelity to intervention Predictors of implementation: Clinician Characteristics: demographics Clinical Outcomes: Posttraumatic Stress Disorder (PTSD) symptoms (*Clinician Administered PTSD Scale* [63]*)* Substance Use (*Addiction Severity Index* [64])	Primary Outcome: Clinicians were able to deliver both therapies with at least adequate fidelity. Predictors of implementation: Clinician Characteristics: Gender - predictive of higher adherence and competence ratings for both ICBT and IAC therapies. Education level - predictive of higher fidelity as session 1 but not session 4. Clinical Outcomes: Fidelity to ICBT at session 4 predicted reductions in alcohol problem severity. Fidelity to IAC at session 4 predicted greater drug severity reductions.
Miller et al. (2004) [65]	MI	Nil	MULTIPLE 2-day Workshop/2-day workshop plus feedback/2-day workshop plus up to 6 individual coaching sessions/2-day workshop, ongoing feedback and up to 6 individual coaching sessions/self-guided	RCT	Participants: Gender: female (50%), Age: 48 years, Education: Master’s Degree or more (85%), Experience: 11 years, Therapeutic Orientation: CBT (48%), 12-step, (26%), humanistic (22%) Treatment Setting: USA	Primary Outcome: Fidelity to intervention Predictors of implementation: Clinician Characteristics: substance use history, self-esteem, attitudes associated with drinking outcomes, temperament	Primary Outcome: The four trained groups had significantly greater gains in fidelity compared to controls. Predictors of implementation: Sustainability - only feedback and coaching) conditions achieved fidelity at follow-up.
Morgenstern et al. (2001) [66]	CBT	Nil	MULTIPLE Didactic, clinical case training workshops, supervision vs. controls	RCT	Participants: Gender: female (65%), Age: 42 years, Ethnicity: Caucasian (72%), African American (21%), Hispanic (7%); Education: Master’s Degree or more (45%) Experience: ‘extensive’ Treatment Setting: USA, Outpatient programs	Primary Outcome: Fidelity to intervention Predictors of implementation: Clinician Characteristics: demographics, beliefs about the nature of alcoholism and substance abuse treatment Clinician evaluation: satisfaction with training and methods, perceived clinical utility, appraised self-efficacy, ideological conflict	Primary Outcome: Positive response to the CBT content and format of the training. Predictors of implementation: Clinician evaluation: Satisfaction with the training as a whole, satisfaction with manualised training method, high perceived clinical utility of CBT. Ideological conflict - little evidence of dogmatism or closed-mindedness.
Rawson et al. (2013) [67]	CBT	Nil	TECHNOLOGY Distance learning through teleconferencing vs. training and coaching in person vs. controls (manual and - hour orientation)	RCT	Participants: Gender: female (75%), Age: 38.1 years, Ethnicity: ‘White’ (36%), ‘Black’ (31%), ‘Coloured’ (19%), other (14%), Education: Bachelor’s degree or more (62.3%) Experience: 7 years Treatment Setting: South Africa, outpatient addiction treatment centres	Primary Outcomes: Fidelity to intervention Knowledge Predictors of implementation: Clinician Characteristics: demographics, training, experience, therapeutic orientation, knowledge, skills in intervention Cost	Primary Outcome: Significant differences found between groups in knowledge and fidelity. Predictors of implementation: Clinician Characteristics: CBT Knowledge - training and coaching in person brought about a significantly greater gain in CBT knowledge. CBT Fidelity - the distance learning and training and coaching in person groups had significantly better skills. Training and coaching in person achieved a higher level of fidelity overall. Cost Comparison: The training and coaching in person condition was most expensive followed by the distance learning and control conditions.
Smith et al. (2012) [68]	MI	Nil	TECHNOLOGY Tele-conferencing supervision (TCS) plus workshop vs. standard tape-based supervision plus workshop vs. workshop alone	RCT	Participants: Gender: female (65%), Age: 44 years, Ethnicity: African American (40%), Caucasian (29%), Latino (26%), other (5%), Education: Bachelor’s degree or more (71%), Treatment Setting: USA, community-based, NIDA	Primary Outcome: Fidelity to intervention Predictors of implementation: Clinician Characteristics: demographics, treatment clinic, years in the field, years in current position	Primary Outcome: TCS plus workshop training increased fidelity, but supervision methods need improvement. Predictors of implementation: Overall, the findings support the importance of providing feedback and supervision after workshop training to improve fidelity, which could potentially be achieved through a TCS format.
Weingardt et al. (2006) [69]	CBT	Nil	TECHNOLOGY Web-based training vs. face-to-face training workshop with identical content vs. delayed training controls	RCT	Participants: Gender: female (55%), Age: 44 years, Ethnicity: Caucasian (56%), African American (21%), Latino (12%), other (10%), Education: Bachelor’s or more (81%), Experience: 7 years Treatment Setting: USA, counsellor outpatient	Primary Outcome: Knowledge Predictors of implementation: Clinician Characteristics: experience, education, familiarity with intervention at baseline	Primary Outcome: Clinicians in both the web-based technology (WBT) and face-to-face workshop conditions showed significant improvement in knowledge compared to clinicians in the delayed training control condition. Predictors of implementation: No significant findings.
Weingardt et al. (2009) [70]	CBT	Nil	TECHNOLOGY Use of web conferencing. Online modules on CBT and group supervision sessions via web conferencing	Randomised trial (randomised to either strong or weak adherence expectations)	Participants: Gender: female (62%), Age: 47 years, Ethnicity: Caucasian (64%), Education: Bachelor’s degree or more (68%), Treatment Setting: USA, counsellor outpatient	Primary Outcome: Knowledge Self-Efficacy Predictors of implementation: Clinician Characteristics: demographics, SUD recovery, familiarity with intervention, work setting, job Burnout	Primary Outcome: Statistically and clinically significant differences in knowledge and self-efficacy were obtained for the web-conferencing group. Predictors of implementation: No significant findings.

*USA* United States of America, *MI* motivational interviewing, *CM* contingency management, , *AA* Alcoholics Anonymous, *NA* Narcotics Anonymous, *TLFB* time line follow back, *PTSD* posttraumatic stress disorder, *CBT* cognitive behavioural therapy, *SUD* substance use disorder, *EBTs* evidence-based treatments, *EBPs* evidence-based practices, *TAU* treatment as usual, *SAMHSA* Substance Abuse and Mental Health Services, NIDA National Institute on Drug Abuse**,** *A-CRA* The Adolescent Community Reinforcement Approach, *P4P* pay for performance, *BMI* brief motivational interviewing, *RCT* randomised controlled trial, *SSL* science to service laboratory, *PAS* provider attitudes scale, *ORCA* Organisational Readiness to Change Assessment, *TC* therapeutic community, *MOC* managing organisational change, *DAT* drug abuse treatment, *TEACH-CBT* Technology to Enhance Addiction Counselor Helping – Cognitive Behavioural Therapy, *MDFT* multi-dimensional family therapy, *MET* motivational enhancement therapy, *MIA:STEP* Motivational Interviewing Assessment: Supervisory Tools for Enhancing Proficiency, ICBT Integrated Cognitive Behavioural Therapy, IAC Individual Addiction Counselling, *TCS* Tele-conferencing supervision, *WBT* Web-based technology

### Treatment settings and participant characteristics of included studies

The majority of studies (16, 80% [39, 42, 45, 47, 54, 59–62, 65, 66, 68–71]) were conducted in the United States of America (USA), outpatient, not-for-profit drug and alcohol services. Alternate settings included one USA adolescent day programme affiliated with the University of Miami Medical School and Jackson Memorial Hospital [55], one outpatient drug and alcohol service affiliated with a university hospital in Switzerland [46], one drug abuse treatment organisation in Peru funded by a US Department of State contract [50], and one involved outpatient addiction treatment centres in South Africa [67]. Study participants were most often female (50–82%) drug and alcohol clinicians, with a mean age ranging from 37 to 48 years. Participants were also mostly Caucasian (50–100% in US studies) and were otherwise African American (14 to 40%), Hispanic (7 to 50%) or some other type of ethnicity (1 to 12.6%). In the South African study participants were also mainly Caucasian (36.4%), with Africans representing 30.8%, 12.6% identifying as “mixed-race”, and 14% Other. Participants commonly held bachelor’s degrees or higher (54 to 100%) and had 3+ to 9.5 years of experience.

### Study designs

Nine (45%) of included studies were randomised controlled trials [59, 61, 62, 65–69], eight (40%) were randomised trials [39, 42, 43, 50, 54, 60, 70, 71](one of which was a subject-by-trial split plot design with repeated measures, [50]), one was a cluster randomised trial [45], one was an interrupted time series design [55], and one was a controlled before-and-after study [47]. Studies varied in terms of the number of participants, the length of follow-up period, the number of addiction services clinicians were sourced from, and the levels of intervention in the approach.

### Types of strategies evaluated

All included studies were concerned with training as an implementation strategy. Approximately one third (*n* = 7) used multiple strategies that involved both passive (e.g. manuals and seminars) and active (e.g. supervision, workshops and champions) approaches to training [47, 59, 62, 65, 66, 68, 71], while 20% (*n* = 4) focused on discrete strategies (e.g. supervision [61], financial incentives [45], booster sessions [50], and workshop only [46]). Another third (*n* = 6) used technological strategies such as teleconferencing and web-based training [42, 54, 67–70]. Three studies (15%) focused on the influence of the intervention context on the uptake of the intervention [39, 55, 60].

### Theories, models and frameworks

Fixsen and colleagues’ [16] conceptualisation of the implementation literature was the most frequently cited (3 of the 20 studies). These studies [59, 60, 67] incorporated Fixsen et al.’s recommendations regarding the importance of training in evidence-based practices through establishing i) program-based advocates, ii) providing adequate feedback and supervision, and iii) developing cost-effective approaches to training and coaching treatment providers. Suggestions from Carroll and Rounsaville [72] were also incorporated in one study [59] specifically in regards to the lack of effective program-based supervision in empirically supported treatments being one of the largest barriers to the implementation of these approaches in clinical practice. While only two studies were guided by Rogers’ [44, 48] argument that individuals are more likely to adopt an intervention after they have an increased knowledge about it and then develop a more favourable attitude towards it, eight (40%) adopted the notion that clinician factors may mitigate the relationship between fidelity to an intervention and patient outcomes [39, 42, 43, 46, 47, 54, 60, 62]. Clinician factors of interest included demographics (e.g. gender, age, experience, education; measured in all of the studies, although only sixteen (80%) reported an intention to evaluate these factors in relation to the implementation, [39, 42, 43, 46, 47, 50, 54, 59, 62, 65–71]), knowledge (3 studies, [67, 69, 70]) and attitudes (6 studies, [39, 43, 46, 54, 59, 65], e.g. beliefs about the origins of addictive behaviour, beliefs about evidence-based treatments (EBTs) or about the intervention itself; learning, confidence and commitment). Factors related to the context of the intervention were the focus of five studies [39, 43, 55, 59, 60], and included organisational factors, organisational readiness for change, and the importance of the context and multilevel approaches. Only two of these studies [55, 60] adopted Simpson’s [56] recommendations about “systemically-oriented” dissemination models, and the evaluation of these efforts in multiple domains, including organisational, clinician and client outcomes. However, two studies [39, 47] used a comprehensive implementation framework. One was entitled “Context-Tailored Training” [39], which is a method of training tailored to the unique challenges of a work setting and the other was the CFIR [47].

The remaining studies drew upon general research or theories that provided a rationale for the training strategies employed. For instance, some identified specific barriers to implementation such as the barrier of limited resources and the challenge of developing cost-effective approaches, (e.g. [5, 73, 74], others presented evidence for the potential uses of technology (e.g. [75])and two studies referenced psychological theories that inform approaches to learning (e.g. [76–80].

### Consolidated framework for implementation research conceptual domains

As can be seen in Table 3, of the 36 subdomains of the CFIR, 32 were evaluated by included studies, although one study [39] mainly contributed to the breadth of coverage. Missing constructs included Intervention Characteristics related to *evidence strength and quality*, Outer Setting constructs including *peer pressure* and *external policies and incentives*, and the Inner Setting construct related to the *relative priority* of the implementation climate. While sixteen (80%, of studies) evaluated Characteristics of Individuals, less than half (9, 45%) measured Intervention Characteristics, and even fewer (4, 20%) measured Inner Setting constructs, with only one study [39] measuring Outer Setting constructs and the Process domain.

**Table 3:** Study findings classified by CFIR domain

	Baer et al. 2009 [39]	Carpenter et al. 2012 [42]	Carroll et al. 2006	Decker and Martino 2013 [43]	Garner et al. 2012 [45]	Gaume et al. 2014 [46]	Helseth et al. 2018 [47]	Johnson et al. 2002 [50]	Larson et al. 2013 [54]	Liddle et al. 2010 [55]	Martino et al. 2008 [59]	Martino et al. 2011 [60]	Marrtino et al. 2016 [61]	Meier et al., 2015 [62]	Miller et al., 2004 [65]	Morgenstern et al. 2001 [66]	Rawson et al. 2013 [67]	Smith et al. 2012 [68]	Weingardt et al. 2006 [69]	Weingardt et al. 2009 [70]
**I. Intervention characteristics**																				
Intervention source							E	E												
Evidence strength and quality																				
Relative advantage	O			E		E	E	E		O						O				
Adaptability	E																			
Trialability	E						E													
Complexity (reverse rated)							E													
Design quality and packaging	O							O					O			O				
Cost	E												O				O			
**II. Outer setting**																				
Patient needs and resources	E																			
Cosmopolitanism	E																			
Peer pressure																				
External policy and incentives																				
**III. Inner setting**																				
Structural characteristics								E	O											
Networks and communications	E																			
Culture								O												
Implementation climate																				
Tension for change	E						E													
Compatibility	E						E													
Relative priority																				
Organisational incentives and rewards	E						E													
Goals and feedback	E						E													
Learning climate	O						E	O		O										
Readiness for Implementation																				
Leadership Engagement	E																			
Available resources	E																			
Access to knowledge and information	E																			
**V. Characteristics of individuals**																				
Knowledge and beliefs about the intervention	O								E							O	E		E	O
Self-efficacy	O			O		O		O								E				O
Individual state of change									E											
Individual identification with organisation	E																			
Other personal attributes	O	O	E	O		O	O	O	E		E			O	O	E	E	E	E	E
**V. Process**																				
Planning	E																			
Engaging																				
Opinion leaders	E																			
Formally appointed internal implementation leaders	E																			
Champions	E																			
External change agents	E																			
Executing	E																			
Reflecting and evaluating	E																			

*E* evaluated, *O* outcome reported

### Implementation, service system and clinical factors evaluated

Almost all implementation outcome measures were concerned with fidelity to the intervention (17, 85%), although three studies measured knowledge [67, 69, 70], two studies measured self-efficacy [70, 81], two studies measured the cost of the intervention [61, 67], two studies measured adherence to the training [39, 55], one study measured supervision integrity [61], and one study measured adoption [47]. Predictors of implementation including clinician characteristics were measured by sixteen (80%) studies and clinician evaluation of the training was measured by four (20%) studies [39, 47, 50, 66]. The most frequently measured clinician characteristics were demographics (such as age, gender, ethnicity, education, experience, prior exposure to the intervention, counselling style or techniques, knowledge and attitudes towards evidence-based practices or the intervention itself, and recovery status). Predictors of implementation related to organisational level factors were measured by five studies [39, 47, 50, 54, 55], covering categories including acceptability, appropriateness, feasibility and penetration. Clinician evaluations of the training largely related to satisfaction with the format, methods, attributes and overall experience of the training, as well as the clinical utility of the training, and one question addressed any ideological conflict experienced. Further questions assessed clinicians’ views of the classroom environment (1) and cultural sensitivity of the training material (1).

Service system outcomes such as location, entry criteria, types of services offered, client to staff ratio, staff turnover, and record data quality, were measured by one study [50].

Clinical outcomes were measured by eight (40%) studies. Substance use was the most frequently measured outcome, followed by retention in treatment. Other clinical outcome measures included emotional and behavioural symptoms, change in motivation and treatment utilisation. In terms of patient characteristics, two studies measured age and gender [46, 71], one of which [71] also measured additional demographics, employment status, admission to the legal system, prior treatment and type of substance.

### Effectiveness of implementation strategies

#### Outcome data by CFIR domain

Outcomes were reported for 9 of the 36 subdomains of the CFIR (see Table 3). Characteristics of Individuals including *other personal attributes* (7 sources, [39, 42, 43, 46, 50, 62, 65])*, self-efficacy* (5, [39, 43, 46, 50, 70])*,* and *knowledge and beliefs about the intervention* (3, [39, 66, 70]), was the domain with most outcome data. Outcome data also related to Intervention Characteristics including *design quality and packaging* (4 sources, [39, 50, 61, 66]), *relative advantage* (3, [39, 55, 66]) and *cost* (2, [61, 68]), and Inner Setting constructs including implementation *learning climate* (3 sources, [39, 50, 55]), *culture* (1, [50]) and *structural characteristics* (1, [54]). Due to the sparse coverage of outcome data relating to CFIR constructs, a more meaningful approach to reporting this information is to discuss the effectiveness of outcomes in relation to implementation factors and their relationships to primary, clinical and service system outcomes.

#### Strategies that effectively enhanced primary outcomes

Effective strategies have been summarised in Table 4. Of the seventeen studies with primary outcomes related to clinician fidelity to the intervention, fourteen achieved positive outcomes (70%), two involved strategies that were somewhat effective [61, 68], and one was not effective [54]. Three of the effective studies also found evidence for increases in clinician knowledge following the implementation [67, 69, 70], one of which found an increase in clinician self-efficacy [70]. A diverse range of methods, strategies and study designs are represented in this sub-group of studies. The majority (60%) evaluated discrete strategies such as the use of local experts trained to provide supervision [43, 59], financial incentives [45], theoretically grounded “booster sessions” [50], web-based training [69, 70], training or supervision via teleconferencing [42, 67], context based interventions [39, 55, 60] and workshop alone (for a brief intervention [46];). The remaining six studies achieving effective outcomes related to fidelity involved strategies with multiple approaches to training. Two of the studies in particular [43, 65] compared passive with active strategies and concluded that active approaches (such as participatory workshops, feedback and supervision) are more effective than passive strategies such as self-study [65] and workshops that provide didactic information only [68]. These conclusions are echoed by the four additional studies of multiple approaches, which included control group comparators. Each of the studies in this group concluded that passive approaches plus supervision are effective ([59, 62, 66, 71], and that coaching workshops [66] or local experts [59] can also add to the effectiveness of the implementation. One study involving multiple strategies and assessing implementation outcomes related to the adoption of the intervention [47] also found evidence for the effectiveness of passive plus active approaches to training. However, clinician factors were found to influence the effectiveness of the implementation over and above the presence of active and passive strategies [42, 43]).

**Table 4:** Factors that effectively enhanced implementation outcomes

	Baer et al. 2009 [39]	Carpenter et al. 2012 [42]	Carroll et al. 2006	Decker and Martino 2013 [43]	Garner et al. 2012 [45]	Gaume et al. 2014 [46]	Helseth et al. 2018 [47]	Johnson et al. 2002 [50]	Larson et al. 2013 [54]	Liddle et al. 2010 [55]	Martino et al. 2008 [59]	Martino et al. 2011 [60]	Martino et al. 2016 [61]	Meier et al., 2015 [62]	Miller et al., 2004 [65]	Morgenstern et al. 2001 [66]	Rawson et al. 2013 [67]	Smith et al. 2012 [68]	Weingardt et al. 2006 [69]	Weingardt et al. 2009 [70]	PERCENTAGE of studies with outcomes
**Outcomes Obtained**	x	x	x	x	x	x	x	x		x	x	x		x	x	x	x		x	x	
Fidelity Outcomes	x	x	x	x	x	x		x		x	x	x		x	x	x	x				82%
Knowledge Outcomes																	x		x	x	18%
Adoption Outcomes							x														6%
Clinical Outcomes			x		x	x		x		x	x	x		x							47%
**Discrete Strategies**		x		x	x			x		x	x	x					x		x	x	**59%**
*Local experts*				x			x				x										*18%*
*Financial incentives*					x																*6%*
*Booster sessions*								x													*6%*
*Web-based training*																			x	x	*12%*
*Teleconferencing*		x															x				*12%*
*Context based*	x									x		x									*18%*
*Workshop alone*						x															*6%*
**Multi-modal approaches**			x	x			x				x			x	x	x					**41%**
**Clinician Characteristics**	x	x		x		x	x	x						x	x						**47%**
Demographics	x	x				x	x							x							35%
*Education*	x	x												x							*18%*
*Gender*						x	x							x							*18%*
Beliefs and Attitudes	x			x		x	x														24%
*Disease beliefs*	x																				*6%*
*Confidence*				x																	*6%*
*Self-efficacy*						x															*6%*
*Intervention efficacy*						x															*6%*
*Intervention Compatibility*							x														*6%*
Learning		x						x													12%
*Vocabulary*		x																			*6%*
*Verbal abstract reasoning*		x																			*6%*
*Affective learning*								x													*6%*
**Organisational Level Factors**	x																				**6%**
Encouraging innovations	x																				6%
Organisational self-efficacy	x																				6%

x = factor significantly related to implementation outcomes. Factors listed here were not measured by all included studies. Percentages refer to the percentage of studies that obtained findings related to a particular factor across studies that achieved implementation outcomes

Outcomes related to clinician characteristics were obtained by eight (40%) of studies and included background demographics, beliefs and attitudes, and learning. With regard to demographics, higher levels of education were associated with higher levels of motivational interviewing (MI) skills and were sustained over time [39], clinicians with no graduate degree experienced the greatest increase in MI spirit following the intervention [42], and clinicians with bachelor’s or master’s degrees were more competent initially but these differences were no longer evident by the end of the training [62]. Gender was found to predict adherence and competence by the end of training in integrated cognitive behavioural therapy (ICBT) [62], young men with male counsellors (and counsellors with more experience) were found to have better outcomes compared to controls [46], and female clinicians delivered contingency management (CM) more frequently at a trend level [47]. Results pertaining to the impact of clinicians’ beliefs and attitudes demonstrated that those with lower initial endorsement of disease belief models had higher levels of MI skills that were sustained at follow-up [39], that confidence was found to be associated with increased competence and adherence [43], that clinicians who viewed themselves as more effective in delivering the intervention and those having higher belief in the efficacy of the intervention also had clients with better outcomes [46], and the negative effect of higher Compatibility (i.e. the perception that the new practice aligns with one’s values, needs, and experiences) on CM adoption was attenuated by the training [47]. In terms of learning, clinicians with low average to average verbal abstract reasoning performances had higher MI Spirit following training than their counterparts [42], and aspects of “affective learning” related to empowerment or confidence were established and maintained following training [50].

At the organisational level, one study found that implementation strategies effectively increased the acceptability of the approach by engendering an openness to the new techniques [39]. More specifically, organisations who encouraged staff to do new things and had higher organisational self-efficacy also had clinicians with higher MI spirit, and agencies with greater openness to new techniques had clinicians who displayed a greater baseline to 3-month MI skill increase. Greater penetration was achieved in two studies [50, 55], which related to organisational decisions to implement the approach with fidelity [50] and an assessment of the programme environment as more controlled, greater clarity of programme expectations being communicated and patient reports of increased autonomy during the implementation [55]. Although one study found increased feasibility of an internet based training course within larger units, whilst training with a treatment manual was found to be more feasible in smaller agencies [54], another study found no relationship between the primary outcome and organisational size and makeup, patient retention or staff turnover [50].

Three studies of effective implementation strategies also conducted evaluations of the training. Clinicians’ appraisals of trainer competency and curriculum content, cultural sensitivity and classroom environment were very positive in one study [50], satisfaction with the training and its methods was high and there was a perceived utility about the intervention in a second study [66], and high satisfaction with the format, trainers and overall experience of training in a third study [39]. Evaluations of the relative costs of the intervention were conducted in two studies [61, 67].

#### Clinical outcomes

Positive patient-level outcomes were obtained by eight (40%) of studies. Specifically, a reduction in substance use was found following the implementation of Brief MI (BMI) workshops [46], a collaborative approach to multi-dimensional family therapy training (MDFT [55];), and the use of manual, workshop and supervision to train clinicians in Integrated CBT [62]. However, there was no change in substance use or treatment utilisation found in Martino et al.’s [61] study of a more cost-effective supervision approach to MI training. One study demonstrated an increase in patient retention following training (workshop and supervision for MI [71];), although the intervention had no significant impact on patient retention in two other studies (cost-effective supervision method [61]; booster sessions, [50]). Change in motivation was observed in patients treated by clinicians trained in motivational enhancement therapy (MET) following training with workshop, supervision and a local expert [59], and emotional and behavioural symptoms associated with problematic substance use decreased after receiving MDFT from clinicians trained via a collaborative approach [55].

#### Service system outcomes

There were no significant service system outcomes reported.

## Discussion

This is the first systematic review of implementation studies in drug and alcohol settings for a range of evidence-based psychosocial approaches. A revealing finding of our review pertains to the lack of utilisation of implementation theories, models and frameworks in substance use specialty care, whereby only two studies used a comprehensive implementation framework [39, 47]. Given the plethora of frameworks, models and theories available (e.g. [11, 14]), the findings of this review suggest that implementation research has been underutilised as a potential guide for implementation research in drug and alcohol settings and substantiates the findings of other recent reviews demonstrating suboptimal use of implementation frameworks [13, 82].

Despite the underuse of implementation research in the development of strategies, there is a general recognition of the necessity of including active as well as passive strategies in SUD treatment research, which is supported by similar findings from both the health and mental health literature (e.g. [18, 83–85]). There are some exceptions to this trend, including studies that focused on the use of discrete strategies (e.g. supervision, booster sessions, technology) or on the influence of the context on implementation outcomes, but the reasoning behind these decisions was more to do with design factors rather than neglect of rigour or the importance of active approaches. While the particular strategies employed in the studies under review here are largely effective and reasonable, when viewed in the context of the CFIR domains, it is evident that several levels of influence are not addressed.

An analysis of results according to the CFIR domains and subdomains revealed that, although Characteristics of Individuals and to some extent Intervention Characteristics are given consideration, Inner Setting, Outer Setting and Process domains are largely neglected, and four subdomains are not assessed by any of the studies. Put simply, influences from within the organisation (e.g. team culture, leadership engagement, the implementation climate), external influences (e.g. patient needs and resources, organisational networks, external policies and incentives), and stages of the implementation process (e.g. planning, executing, reflecting and evaluating) are important aspects of the multi-level nature of implementing evidence-based practice and warrant further study in drug and alcohol settings. More recently, other implementation frameworks have been utilised more comprehensively to drive implementation strategy conceptualisation, strategies, measurement, and movement through phases of the implementation process that might address some of the gaps in the CFIR such as the Exploration, Preparation, Implementation, Sustainment (EPIS) framework [86]. The EPIS was used effectively to enhance service delivery amongst justice-involved youth accessing complex, multi-agency systems [14].

The literature suggests that clinician factors and (to a lesser extent) organisation level factors moderate the effectiveness of implementation over and above the presence of active and passive strategies. When implementing a training programme designed to upskill drug and alcohol clinicians in an evidence-based approach to practice, important factors to consider include certain demographics, clinician beliefs and attitudes, and modes of learning. Specifically, it may be important to modify training according to a clinician’s level of education (since highly educated clinicians may need a more challenging regime in order to make significant gains in proficiency) or verbal abstract reasoning performance (since those who are low on this trait may experience greater gains following the intervention), and to be aware of the influence that clinician gender and patient gender may have on the implementation outcome. It is also interesting that clinician beliefs and attitudes such as lower endorsement of disease belief models, higher confidence, more belief in one’s ability to deliver the intervention effectively, and higher belief in the efficacy of the intervention may moderate clinician fidelity to the intervention. Important organisational factors contributing to improved implementation outcomes might include organisational contexts in which staff are encouraged and supported to apply new practices, higher organisational self-efficacy, and greater openness to new techniques. It should also be noted that an overall strength of this implementation research into the transfer of evidence-based practices in drug and alcohol settings is the finding that three-quarters of the implementation strategies being evaluated were effective.

Limitations of this review surround the challenges of synthesising and comparing information derived from a diverse range of implementation strategies, clinical interventions, study conditions, and types of outcomes. For instance, drawing comparisons across studies that included discrete versus multi-modal strategies versus context-driven strategies, and across studies with different combinations of multi-modal strategies was problematic. Additionally, the fact that almost all studies were based in the US makes the review mainly applicable to that context. Outcomes were also mainly associated with clinician fidelity to the intervention, but were sometimes concerned with knowledge, self-efficacy, adoption and even supervisor fidelity. While the use of the EPOC data form, the conceptual model of Proctor [9] and the CFIR [11] assisted with teasing out these inconsistencies, they are still lacking empirical validation [87]. Lastly, our assessment of risk of bias and the inclusion of eligibility criteria that ensured only studies with rigorous designs became included in the review may have resulted in underreporting of findings related to CFIR domains due to space limitations (as mentioned by [35]) and the exclusion of studies that provide rich information about the implementation but have alternate designs. The exclusion of criminal justice settings might also be seen as a limitation in that certain studies seeking to integrate community-based care with justice settings may have been relevant.

## Conclusion

This review contributes to the growing body of literature on the implementation of evidence-based practice in drug and alcohol settings, which may well have expanded since this review was conducted. It has demonstrated that particular strengths of the implementation research in these settings include the effectiveness of strategies employed, the broad recognition of the importance of understanding clinician characteristics and (to a lesser extent) intervention characteristics impacting the implementation outcomes. There is also some evidence for the mitigating effects of certain demographics, beliefs and attitudes, and modes of learning. On the other hand, this review has revealed that implementation frameworks have been underutilised and important levels of influence have been overlooked (e.g. organisational factors, external forces impacting the implementation, and an understanding of the effects of the process of the implementation). There is a need for determinant and process frameworks as well as integrating evaluation and outcomes frameworks in order to foster further development of approaches to improving implementation science in drug and alcohol service settings.

## Supplementary Information

**Additional file 1.** PRISMA Checklist. Checklist of the Preferred Reporting Items for Systematic Reviews and Meta-Analyses Protocols (PRISMA-P).

**Additional file 2.** Example Study Search. Details of concepts and search terms used in MEDLINE.

## Data Availability

Not applicable.

## Abbreviations

USA: United States of America
MI: Motivational interviewing
CM: Contingency management
AA: Alcoholics Anonymous
NA: Narcotics Anonymous
TLFB: Time line follow back
PTSD: Posttraumatic stress disorder
CBT: Cognitive behavioural therapy
SUD: Substance use disorder
EBTs: Evidence-based treatments
EBPs: Evidence-based practices
TAU: Treatment as usual
SAMHSA: Substance Abuse and Mental Health Services
NIDA: National Institute on Drug Abuse
A-CRA: The Adolescent Community Reinforcement Approach
P4P: Pay for performance
BMI: Brief motivational interviewing
RCT: Randomised controlled trial
SSL: Science to service laboratory
PAS: Provider attitudes scale
ORCA: Organisational Readiness to Change Assessment
TC: Therapeutic community
MOC: Managing organisational change
DAT: Drug abuse treatment
TEACH-CBT: Technology to Enhance Addiction Counselor Helping – Cognitive Behavioural Therapy
MDFT: Multi-dimensional family therapy
MET: Motivational enhancement therapy
MIA:STEP: Motivational Interviewing Assessment: Supervisory Tools for Enhancing Proficiency
ICBT: Integrated cognitive behavioural therapy
IAC: Individual addiction counselling
TCS: Tele-conferencing supervision
WBT: Web-based technology
